# Uremic encephalopathy with isolated brainstem involvement revealed by magnetic resonance image: a case report

**DOI:** 10.1186/s12883-017-0936-9

**Published:** 2017-08-08

**Authors:** Li-jing Jia, Zhen-zhen Qu, Xue-qian Zhang, Yu-juan Tian, Ying Wang

**Affiliations:** 1 Department of Neurology, The Second Hospital of Hebei Medical University, Hebei Shijiazhuang, 050000 People’s Republic of China; 2 Institution of Cardiocerebrovascular Disease, Hebei West Heping Road 215, Shijiazhuang, 050000 People’s Republic of China; 3 Neurological Laboratory of Hebei Province, Hebei Shijiazhuang, 050000 People’s Republic of China

**Keywords:** Uremic encephalopathy, Magnetic resonance images, Brainstem, Renal failure, Posterior reversible encephalopathy syndrome

## Abstract

**Background:**

Uremic Encephalopathy (UE) is a neurological complication associated with acute or chronic renal failure. Imaging findings of UE may present involvement of the basal ganglia, cortical or subcortical regions, and white matter. We report a rare case of UE caused by neurogenic bladder with isolated brainstem involvement revealed by magnetic resonance imaging (MRI). Immediate therapy resulted in full recovery of neurological signs and changes on MRI.

**Case presentation:**

A 14-year-old Han Chinese woman with a history of chronic renal failure caused by neurogenic bladder. On admission, she was unconscious and her pupils presented different sizes, while her vital signs were normal. MRI showed high signal in the dorsal pontine base and in the mid brain on fluid-attenuated inversion-recovery (FLAIR) imaging and on T2-weighted imaging while the signal was normal on diffusion-weighted images (DWI). Blood analysis revealed renal failure and acidosis. After urinary retention treatment and acidosis correction, the patient soon recovered. Follow-up MRI 2 months after the discharge revealed complete resolution of UE in the brainstem.

**Conclusion:**

We reported a rare case of a patient with UE that had unusual imaging manifestations for whom timely diagnosis and treatment assured recovery.

## Background

Uremic encephalopathy (UE) may result from multiple disorders associated with acute or chronic renal failure and is characterized by acute or subacute onset of reversible neurological symptoms. There are 3 patterns of imaging findings in patients with UE: involving the basal ganglia, cortical or subcortical regions, and white matter [1]. Cortical involvement of UE is a category of posterior reversible encephalopathy syndrome (PRES) and basal ganglia involvement is rare [1, 2], the white matter involvement is limited to case reports [3, 4]. We came across a reported case of UE caused by neurogenic bladder that presented isolate involvement of the brainstem on magnetic resonance imaging (MRI). To our knowledge, only two other reported cases had MRI findings similar to this patient [5, 6]. We report a rare case of UE with unusual imaging manifestation to encourage timely diagnosis and treatment of this condition.

## Case presentation

**Fig. 1:**
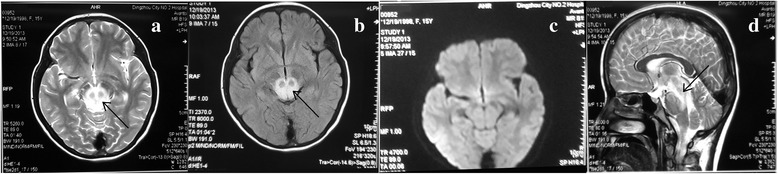
Brain MRI after the symptom onset. T2-weighted imaging (T2WI) and fluid-attenuated inversion-recovery (FLAIR) imaging revealed isolated hyperintense signals in the dorsal part of the pons and the midbrain in axial view (**a**, **b**) and sagittal view (**d**). Diffusion-weighted imaging (DWI), however, showed normal intensity (**c**)

A 14-year-old Han Chinese woman with a history of neurogenic bladder for more than 10 years was admitted to our Department because of coma for 12 h. The patient complained for diplopia without fever or diarrhea 4 days before her coma with no incentive. She had no limb weakness and was treated with Vitamin B1and mecobalamine. Her previous medical history included neurogenic bladder caused by spina bifida occulta for 12 years and hydronephrosis diagnosed 1 year before. Sometimes she had urine retention, but she received no treatment for the neurogenic bladder except for urethral catheterization occasionally. She had no seizures and no fever. On admission, physical examination revealed that she was unconscious. The pupils did not react to light and presented different sizes in each eye (left pupil diameter, 2.5 mm; right pupil diameter, 4 mm). On stimulation, she could move her 4 limbs spontaneously. There were exaggerated deep tendon reflexes over both legs and the right Barbinski sign was positive. However, her vital signs were stable: Blood Pressure (BP) was 120/81 mmHg; pulse rate, 76 beats/min; body temperature, 36.5 °C; breath, 12 times/min. Non-contrast brain computed tomography (CT) after the onset of her coma revealed diffuse brain stem hypodensity. Brain MRI acquired 4 h after the coma onset in the county hospital showed high signal at the dorsal part of the pontine base and the mid brain on fluid-attenuated inversion-recovery (FLAIR) imaging and T2-weighted imaging. However, diffusion-weighted images presented normal signal (Fig. 1). The non- contrast abdominal CT preformed on the next day of admission showed chronic obstructive uropathy with a distended bladder, severe hydronephrosis and dual renal atrophy.

Blood analysis immediately after admission revealed renal failure [Urea nitrogen: 23.87 mmol/L, Creatinine: 566.8 μmol/L], and anemia [RBC: 2.5 × 10^12^/L, HGB: 79 g/L]. Arterial blood gas analysis revealed metabolic acidosis [pH 7.09 (7.35–7.45)]. Liver function was normal.

**Fig. 2:**
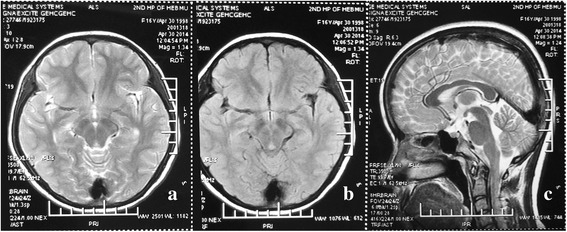
Follow up magnetic resonance imaging (MRI) showed significant decrease in signal intensity in the brainstem on axial T2-weighted imaging (T2WI) (**a**), axial fluid-attenuated inversion-recovery (FLAIR) imaging (**b**), and sagittal T2WI (**c**)

The patient was diagnosed with UE due to chronic renal failure and metabolic acidosis. She was immediately treated with 200 ml of bicarbonate at 5% conducted by quick intravenous injection, followed by 100 ml intravenous injection of bicarbonate at 5% once a day and 1.0 g of bicarbonate administered orally three times per day for 3 days. Simultaneously, she underwent urethral catheterization to relieve the urine retention. She was revived without diplopia 3 days after admission and the reviewed blood analysis after urethral catheterization showed that her renal function was returning to a normal level (Urea nitrogen: 13.10 mmol/L, Creatinine: 294 μmol/L); hence, dialysis was not necessary. She was treated with hypodermically administered erythropoietin 3000u twice a day, and ferrous sulfate 0.3 g orally thrice a day for the anemia. She was discharged 14 days after the admission without diplopia and any mental disturbance. She was still with the ureteral catheter because of the hydronephrosis. Two weeks after the discharge, the catheter was removed when abdomen ultrasonic showed decreased hydronephrosis. Follow-up MRI performed 2 months after the discharge revealed complete resolution of the brainstem UE (Fig. 2). Reviewed blood analysis after 2 months of discharge showed that her renal failure still persisted (Urea nitrogen: 13.10 mmol/L, Creatinine: 190.3 μmol/L). However, she had no neurological disturbance or urinary retention. She underwent the comprehensive bladder-retraining program to avoid urinary retention and her renal function was regularly assessed once a month.

## Discussion and conclusions

Clinical manifestations of UE are variable and alterations in brain images play an important role in the diagnosis of the condition. Unusual for typical UE imaging manifestations, the imaging findings of our patient showed isolated brainstem hyperintensity on FLAIR and T2WI without basal ganglia and cortical or subcortical involvement. As opposed to the typical posterior reversible encephalopathy syndrome (PRES), central-variant posterior reversible encephalopathy syndrome had been described as brain stem or basal ganglia involvement that spares the cerebral cortex and subcortical white matter [7], which had similar imaging features with our patient. Approximately one-third of PRES cases are reported to have involved the brainstem and basal ganglia, accompanied by involvement of the parieto-occipital regions [8] and only 4% of these patients did not have cortical involvement [7]. As for UE, there were few reports on the isolated brain stem involvement detectable through MRI. Although the brainstem was involved and the patient presented severe symptoms, timely and accurate therapy resulted in clinical recovery.

Although the pathophysiological changes are not fully understood, UE and PRES share similar clinical and MRI features. The pathophysiology of PRES is thought to be the dysfunction of vascular autoregulation whereas UE is likely caused by the effects of neurotoxic compounds [1]. Regardless the reason, the breakdown of the blood-brain barrier is the key factor of the imaging manifestations [9]. MRI predominantly reveals vasogenic edema but cytotoxic edema has been reported in UE and PRES [1, 3, 4]. The imaging manifestations of our patient showed hyperintensity on T2WI and FLAIR but normal intensity on DWI, which represented vasogenic edema. We assumed that this image finding may be caused by the breakdown of the blood-brain barrier related to the uremic toxin or acidosis. When the acidosis was corrected (PH 7.42) and the renal failure decreased (Urea nitrogen: 14.6 mmol/L (1.7–7.1 mmol/L), Creatinine: 294 μmol/L (0.1–136 μmol/L), after the urinary obstruction was relieved 3 days after the admission, the clinical symptoms of the patient resolved. Follow-up images acquired 2 months after the discharge showed complete resolution of the brainstem even though the renal failure was not completely recovered (Urea nitrogen: 13.10 mmol/L, Creatinine: 190.3 μmol/L). However, we are not able to comment on why the lesion was located in the brain stem without cortical or basal ganglia involved.

The brainstem CT and MRI findings in our patient should be differentiated from Wernicke’s encephalopathy, central pontine myelinolysis, and infectious brainstem encephalitis. The lack of no increased signal in periaqueductal regions and medial thalami make Wernicke’s encephalopathy unlikely. Infectious encephalitis was ruled out, because there was no fever and other signs of infection. The reversibility of symptoms and imaging findings coupled with a negative history of rapid correction of hyponatremia makes central pontine myelinolysis less likely.

UE in renal failure is reversibly associated with hemodialysis or peritoneal dialysis, which removes neurotoxic compounds. Regarding our patient, her renal failure was caused by neurogenic bladder related to spina bifida occulta. It is reported that among nearly 60,000 patients with neurogenic bladder, the incidence of lower urinary tract infections was 29–36%, urinary retention was 9–14%, and urinary tract obstructions was 6–11% [10]. Our patient had urinary retention and urinary tract obstruction and she presented UE when the renal function got very severe. After the urinary retention was relieved by urethral catheterization and the acidosis was corrected, the renal function improved and the clinical manifestations were recovered without dialysis.

In summary, MRI manifestations are important for the diagnosis of UE. Neurotoxic compounds and metabolic acidosis may play roles in the pathophysiology of this disease. We reported a rare case of a patient with UE that had isolated brainstem vasogenic edema detectable through MRI, assuring timely diagnosis and treatment of the condition.

## Abbreviations

MRI: Magnetic resonance imaging
PRES: posterior reversible encephalopathy syndrome
UE: uremic encephalopathy, renal failure
